# Oviposition Deterrent Activity of Fungicides and Low-Risk Substances for the Integrated Management of the Olive Fruit Fly *Bactrocera oleae* (Diptera, Tephritidae)

**DOI:** 10.3390/insects13040363

**Published:** 2022-04-07

**Authors:** Ilaria Checchia, Corrado Perin, Nicola Mori, Luca Mazzon

**Affiliations:** 1 Department of Biotechnology, University of Verona, Villa Lebrecht, Via della Pieve 70, 37029 San Pietro in Cariano, Italy; ilaria.checchia@univr.it (I.C.); corrado.perin@univr.it (C.P.); nicola.mori@univr.it (N.M.); 2 Department of Agronomy, Food, Natural Resources, Animals and Environment (DAFNAE), University of Padova, Viale dell’Università 16, 35020 Legnaro, Italy

**Keywords:** ovipositional behavior, repellency, plant biostimulants, sustainable agriculture

## Abstract

**Simple Summary:**

The olive fruit fly *Bactrocera oleae* is a very common pest infesting olive orchards wherever they are cultivated, representing the greatest threat to olive production and oil quality. Although broad-spectrum insecticides are often used to protect olive crops against *B. oleae*, there is increasing concern about their effects on the environment and human health. An important tool in integrated olive fly management could be the use of products with a repellency and oviposition deterrence effect. This research yielded experimental evidence of significant oviposition deterrent activity on the olive fly as side effects of substances used in olive growing such as fungicides or plant biostimulants, highlighting the potential use of these products in *B. oleae* management.

**Abstract:**

The control of *Bactrocera oleae* is fundamental to decreasing the significant production loss in olive cultivation. However, traditional containment based on the use of synthetic insecticides has been encountering serious limitations due to their negative effect on human health and the environment. Within the scope of integrated olive fly management, the use of products with repellency and oviposition deterrent activity might represent a more eco-friendly solution. In this study, we tested the oviposition deterrent activity of some commercial formulations already used in olive tree crops as fungicides (copper oxychloride, dodine, mancozeb, pyraclostrobin and difeconazole) and plant bio-stimulants (tannins, clay, flavonoids and a zinc-copper-citric acid biocomplex). The trials were conducted testing the oviposition behavior of mated olive fly females in both choice and no-choice assays. Our results showed that most of the substances have affected the ovipositional activity of the olive fly, except for difeconazole. Moreover, some products (copper oxychloride, flavonoids and tannins) have proven to differently influence the flies’ oviposition comparing the two tests. The repellent effect of these commercial products should be further studied to prove whether the repellency was due either to the active ingredient or to the co-formulants, and to assess their effect in the open field.

## 1. Introduction

The olive fruit fly *Bactrocera oleae* (Rossi) (Diptera, Tephritidae) is a very common pest infesting olive orchards wherever they are cultivated [1], representing the greatest threat to olive production and oil quality worldwide [2].

Traditionally, control of *B. oleae* infestations has been successfully achieved through chemical insecticide cover sprays based on curative activity. However, the insecticides have serious limitations due to their toxic effects on human health, the presence of residues in the olive fruit and oil, the development of insect resistance and the negative impact on non-target arthropods [3,4,5,6,7,8]. In addition, according to the EU Directive 128/2009 on the sustainable use of pesticides, the recent EU Regulation 2019/1090 banned the use of the organophosphate dimethoate, the most applied insecticide for olive fly infestation control, triggering the need to introduce alternative strategies for control of the pest. 

Current available alternatives to broad-spectrum insecticides against the olive fruit fly are represented by preventive attract-and-kill methods, mass trapping devices and the use of biological control agents (BCA) [9] whose performances were already demonstrated in the field even though they vary across different geo-environmental conditions, ecological balances and the level of pest pressure [10,11,12,13,14]. One more alternative is represented by the sterile insect techniques (SIT) whose efficacy and economic viability depend on several factors such as the mass-rearing process, the selection of a suitable strain and their field performance [15]. Another important tool in integrated fruit flies’ management is pest behavior manipulation [16] using products with a repellency and oviposition deterrence effect. For example, one of the pest management approaches is the push-pull technique that is based on the exploitation of insect selection behavior [17,18]. The mechanisms involve a variety of chemical, visual and tactile signals to detect and accept the fruit hosts [19]. The chemotactic repulsion, olfactory repulsion and inadequate odor might be exploited to hinder the adults’ recognition of the fruit [20]. In this context, the repellent and oviposition deterrent effects might be characteristics of many substances used in olive cultivation even though they were not formerly studied for targeting such effects. For instance, research efforts with Mediterranean fruit flies, *Ceratitis capitata* have often focused on the deterrent effects of plant essential and vegetable oils [21] or fruit marking pheromones [22,23]. In *Bactrocera tau* (Walker) some plant products, bio-pesticides and clay showed oviposition deterrence and egg hatching inhibition [24]. Moreover, in the case of *B. oleae,* salts copper and dust had a repellent effect [25,26]. The entomopathogenic fungi *Beauveria bassiana* is used for its pathogenicity on olive fruit fly pupae and adults [27], but it has also been demonstrated that *B. bassiana* conidia impaired the ability of *C. capitata* to detect the stimuli in the fruit recognition process [28].

Hitherto, little systematic research has been conducted to assess the possible effects on *B. oleae* of current formulations developed for disease control such us fungicides against olive knot (*Pseudomonas savastanoi* pv. *savastanoi*), peacock spot (*Spilocaea oleaginea*), Botryosphaeria blight (*Botryosphaeria dothidea*, conidial stage: *Fusicoccum* sp.), olive anthracnose (*Colletotrichum* spp.) or substances to improve the yield and quality of the harvest as well as the resistance to and recovery from different types of abiotic stress (plant biostimulants).

In this study, we investigated under laboratory conditions the effect of some commercial formulations used in olive cultivation on the ovipositional behavior of the olive fruit fly with the aim of considering such beneficial ‘side-effects’ in integrated *B. oleae* control management.

## 2. Materials and Methods

### 2.1. Bactrocera oleae Rearing

Olive fly pupae were collected during November 2020 from oil mills in the Veneto Region (Northern Italy) and routinely within 24 h transferred to the laboratory. Then, to obtain same-aged cohorts, emerged flies were reared in 30 × 30 × 30 cm^3^ net cages in a growing chamber INCOLD^®^ (INCOLD S.p.A, Rovigo, Italy) at 23 ± 2 °C, 65 ± 10% RH and 16:8 (L:D) h photoperiod.

Female and male flies were reared in the same cage and were fed on a dry diet consisting of sugar and yeast extract (Sigma-Aldrich, Burlington, MA, USA) (4:1). Water was constantly available on a sponge wick and refreshed every 7 days.

### 2.2. Commercial Formulation Applications 

We investigated the repellency and oviposition deterrent effects on *B. oleae* of commercial formulations used in olive tree crops as fungicides (copper oxychloride, dodine, mancozeb, pyraclostrobin and difeconazole) or plant biostimulants (tannins, clay, flavonoids and a zinc-copper-citric acid biocomplex) (Table 1). Their activity was compared with those of the entomopathogenic fungi *B. bassiana,* the deterrent effect of which is known in fruit flies [28]. The different substances were investigated at field concentration rate. 

For all the experiments, we used ripe olives, cultivar “Casaliva”, collected from an untreated olive orchard in Veneto Region (Bardolino, VR, 45°31′37.4″ N 10°44′37.0″ E). Fruits were manually collected and transferred to the laboratory within 24 h. Olives were stored at 7 °C for up to 2 months.

Each olive in the experiments was treated by dipping it in the different solutions, then letting it dry at room temperature on a hydrophobic surface under a fume hood. Untreated olives were immersed in distilled water as control. 

The experiments were conducted under laboratory conditions at 20 °C ± 2 and 50–60% RH. At the end of each trial, the olives were carefully inspected under a binocular stereomicroscope in order to detect fertile (holes with eggs) and sterile (holes without eggs) stings. Fertile and non-fertile stings were recognized by making shallow cuts using a scalpel in correspondence to the stings and assessing the presence/absence of an egg.

### 2.3. No-Choice Oviposition Assay

Two-week old mature adults (9 females and 2 males) were gently caged in a plastic box (15 w × 11 d × 6 h cm^3^) covered by a wire mesh on the top to allow aeration. The flies were exposed to 20 olives for 24 h. Olives were either treated with one of the formulates or untreated (control) and placed on the cage floor. Each cage with 20 olives represented a test unit (a repetition). The treatment related to *B. bassiana*, copper oxychloride, tannins, dodine, flavonoids and zeolite was repeated 8 times (8 cages per treatment); the zinc-copper-citric acid biocomplex was repeated 6 times (6 cages per treatment), whereas mancozeb, difeconazole and pyraclostrobin were repeated 4 times (4 cages per treatment). The control treatment was repeated 13 times (13 cages). The difference in the number of repetitions was due to the different availability of flies when the trials were conducted. Water was constantly available through a 2 mL tube filled with absorbent cotton and tap water, whereas food was provided through a drop of mixture of sugar and yeast flakes in a ratio 4:1 on the top of each cage. At the end of the period (24 h), olives were carefully collected and checked for eggs presence.

### 2.4. Dual-Choice Oviposition Assay

One mature female *B. oleae* fly (2 weeks after emergence) was caged for 4 h in a wire mesh box (12 w × 6 h × 7 d cm^3^) and exposed to two olives (one treated and one not). Olives were placed on the bottom of the box avoiding contact with each other and keeping a minimum distance of 3 cm. Neither water nor food were left available during the test periods. Each cage represented a repetition. For each substance under test, at least 30 replicates were done. At the end of the period (4 h), olives were carefully collected and checked for the presence of eggs and stings in both treated and untreated olives.

### 2.5. Statistical Analyses

For the statistical analyses, R software (R 4.0.3) [29] was used in the RStudio environment. In both oviposition and choice tests, data were not normally distributed (Shapiro–Wilk test, *p* < 0.05), thus generalized linear models and non-parametric tests were used to evaluate differences.

In no-choice oviposition assay, data were analyzed using the negative binomial regression model (MASS package) with the function: glm.nb. A negative binomial distribution was adopted since the data were slightly overdispersed. The response variable was the total number of eggs for each cage and the explanatory variable was the treatment. We checked the model for overdispersion and residual distribution using the DHARMa package.

In dual-choice oviposition assay, the number of eggs in the treated olives was compared to the number in the untreated ones (control) using the paired samples Wilcoxon test with the function wilcox.test.

The Kruskal–Wallis rank test was used to compare treatments of dual-choice test with the function kruskal.test. *p*-values were corrected using the Tukey’s method. Statistical significance was established for α < 0.05.

## 3. Results

### 3.1. No-Choice Oviposition Assay

**Figure 1:**
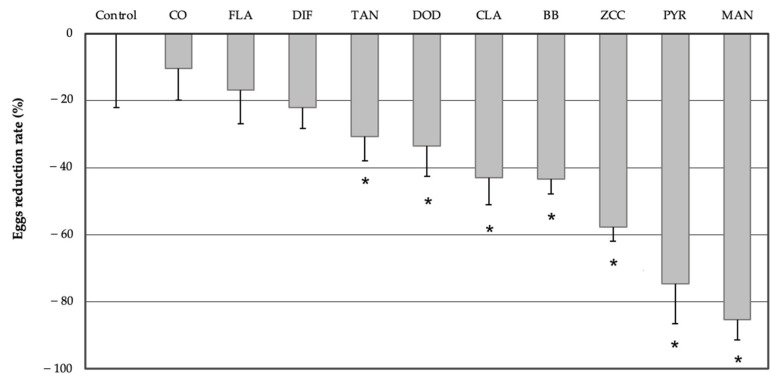
Eggs reduction rate (%) in treated olives compared to the control (set as zero). CO: copper oxychloride, FLA: flavonoids, DIF: difeconazole, TAN: tannins, DOD: dodine, CLA: clay, BB: *B. bassiana*, ZCC: zinc-copper-citric acid biocomplex, PYR: pyraclostrobin, MAN: mancozeb, compared to the control. * *p* < 0.05, significant differences compared to the control (negative binomial model). Bars indicate the negative standard error.

A significant deterrent effect on oviposition was observed for tannins (z = −2.219, *p* = 0.026), dodine (z = −2.457, *p* = 0.014), clay (z = −3.347, *p* = 0.001), *B. bassiana* (z = −3.397, *p* = 0.001), zinc-copper-citric acid biocomplex (z = −4.149, *p* < 0.01), mancozeb (z = −5.171, *p* < 0.01) and pyraclostrobin (z = −4.680, *p* < 0.01). These products significantly reduced the number of laid eggs (Figure 1) compared to the control (untreated).

The analyses of deviance from the negative binomial model testing the effect of treatments on the number of eggs reported the following values: χ²: 28.27657, df: 10 and *p*-value: 0.00163.

### 3.2. Dual-Choice Oviposition Assay

**Figure 2:**
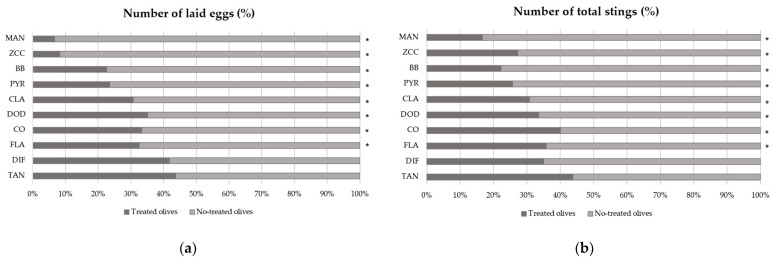
Effects of treatments on the preference for oviposition of *B. oleae* evaluated by counting (**a**) number of laid eggs (%) and (**b**) number of total stings (sterile stings plus stings with laid eggs) in treated and untreated olives. MAN: mancozeb, ZCC: zinc-copper-citric acid biocomplex, BB: *B. bassiana*, PYR: pyraclostrobin, CLA: clay, DOD: dodine, CO: copper oxychloride, FLA: flavonoids, DIF: difeconazole, TAN: tannins. The results are the summed values of thirty replicates in independent repeats. * *p* < 0.05, significant differences compared to the control (paired samples Wilcoxon test).

Most of the products influenced fly’s behavior in terms of oviposition preference (Figure 2). A higher number of eggs and total stings (sterile holes + holes with eggs) were reported in the untreated olives. This result was observed for *B. bassiana* (t = −3.567, *p* = 0.001 and t = −4.472, *p* < 0.01 as concerns laid eggs and total stings, respectively), copper oxychloride (t = −2.386, *p* = 0.020; t = −2.153, *p* = 0.036), dodine (t = −2.657, *p* = 0.010; t = −3.976, *p* < 0.01), flavonoids (t = −2.468, *p* = 0.017; t = −2.439, *p* = 0.019), clay (t = −2.375, *p* = 0.025; t = −2.913, *p* = 0.007), zinc-copper-citric acid biocomplex (t = −2.699, *p* = 0.010; t = −3.081, *p* = 0.004), pyraclostrobin (t = −2.282, *p* = 0.035; t = −3.775, *p* = 0.001) and mancozeb (t = −3.980, *p* < 0.001; t = −4.729, *p* < 0.001). The two products which did not seem to statistically influence the oviposition preference were tannins (t = −1.370, *p* = 0.101; t = −1.721, *p* = 0.090) and difeconazole (t = −0.809, *p* = 0.429; t = −2.051, *p* = 0.055).

The Kruskal–Wallis test did not determine significant differences among treatments for either eggs or total stings (χ²_df = 10_ = 9.4879, *p* = 0.486 and χ²_df = 10_ = 15.262, *p* = 0.123, respectively).

## 4. Discussion

An important tool in integrated olive fly management could be the use of products with a repellency and oviposition deterrence effect. This research showed experimental evidence of significant oviposition deterrent activity on olive fly as side effects of substances used in olive growing, such as fungicides or plant biostimulants.

Regarding fungicides, dodine, mancozeb and pyraclostrobin had a significant deterrent action in reducing the egg laying rate compared to the control, and significantly influenced the oviposition preference in the choice test. Interestingly, among these products, generic side effects in the reduction of density and oviposition of predatory mites were reported only for mancozeb [30], whereas, to our knowledge, no cases are known about the deterrent side effect of these commercial products. Further investigation is needed to understand whether repellency was due either to the active ingredient or to the co-formulants.

In our study, copper oxychloride had a significant deterrent effect only in the choice test; the non-significant egg reduction rate (%) compared to the control observed in the oviposition assay (no-choice) is partially in contrast with other studies showing the efficacy of copper formulations in preventing *B. oleae* attack on olives [25,26]. However, continuous exposure to substances can cause flies to exhibit an increased tendency to lay eggs in marked fruit and in general seems to reduce sensitivity (presumably through habituation or sensory adaptation) [31].

Indeed, it is commonly known that copper can play an important role as oviposition deterrent [26,32]. Furthermore, it is supposed that copper can play an antimicrobial effect [33] on the fruit surface, causing a reduction of those bacterial compounds that make the olive attractive to the fly for oviposition [33]. Based on its antibacterial properties, attention has recently focused on the effectiveness of copper as a bactericide against the primary bacterial symbiont *Candidatus* Erwinia dacicola, thereby interrupting the symbiosis with *B. oleae* essential for its fitness [34,35]. Finally, the treatment based on difeconazole did not influence either the female action to search the fruit or oviposition.

Concerning plant bio-stimulants, our work showed for the first time that the zinc-copper-citric acid biocomplex (Dentamet^®^) significantly reduces both oviposition rates and the total number of laid eggs in treated olives. This commercial product was previously shown to inhibit growth of *Xylella fastidiosa*, reducing the severity of symptoms related to this pathogen in olive trees [36]. Moreover, a recent work showed the suppressive effect caused to brown marmorated stink bug (*Halyomorpha halys*) nymphal survival by exposure to the antimicrobial activity of Dentamet^®^, as a consequence of interrupted acquisition of the symbiont *Pantoea carbekii* [37]. Beyond the new finding about the deterrent activity of Dentamet^®^, our study showed that clay (zeolite) reduces the oviposition rates, which is commonly known and presumably due to the physical barrier on the olive surface as reported for other clay formulates such as kaolin powder and bentonite dust [26,38,39].

Flavonoids (Propolis), similarly to copper oxychloride, did not cause a significant egg reduction rate (%) in the oviposition assay (no-choice), but affected the ovipositional behavior of *B. oleae* in the choice test. The efficacy of flavonoids to restrain the olive fruit fly infestation percentage was previously reported also in experimental fields [40], and was already demonstrated against the melon fruit fly *Bactrocera cucurbitae* (Coquillett) where application in small pieces of pumpkin reduced oviposition by females under choice as well as no-choice conditions [41].

Tannins reduced the numbers of *B. oleae* eggs laid in treated olives confirming the evidence obtained on *B. cucurbitae* with phenolic compounds that effectively reduced egg laying and the mean number of ovipunctures in choice and no-choice tests on the treated substrate [42].

The entomopathogenic fungi *B. bassiana* is largely used in organic orchards to control *B. oleae* infestation [43]. In addition to its pathogenicity on adults [27], our data showed that treatments with *B. bassiana* preparations caused an oviposition deterrent effect in comparison to the untreated control, confirming the results observed in *C. capitata* [28]. The inhibitory effect could be due to volatile organic compounds released by the fungus [44] or to the physical and biochemical properties of conidia that might interfere with the ability of females to detect fruit-derived stimuli, such as odors and humidity content [28].

Further trials should be established to evaluate whether the products tested in this study perform their deterrent activity also when applied in an open field context.

## 5. Conclusions

In conclusion, our results bring to attention the significant oviposition deterrent activity of some substances generally used in olive cultivation, such as fungicides and plant biostimulants. During the *B. oleae* flight period, on average, 3−4 fungicide applications and 1–2 plant biostimulant applications are carried out in olive growing areas against the main diseases and to improve the yield and quality of the harvest [45], respectively. Choosing the proper products in the right period, it is possible to exploit both the target effect and deterrent action against *B. oleae* oviposition for each product. Considering that against olive knot, peacock spot, olive anthracnose chemicals were applied in autumn and spring [46,47], the fungicide sprayings could have preventive side effects on olive fruit flies’ summer generations. Moreover, for each fungicide, it is necessary to consider the limitations of use (maximum number of applications or doses) and for copper salts the limitations of active ingredient amount per hectare and per year in organic agriculture [48,49].

Moreover, the repellent effect could be integrated in a push-pull strategy involving the action of stimuli that make the protected fruits unsuitable to the fly that is at the same time attracted by a luring source (e.g., trapping devices) [50].

Some of the substances that were reported to exert an ovideterrent effect are also known for their antimicrobial activity (e.g., tannins, dentamet, copper oxychloride, flavonoids and dodine). It would be interesting to investigate whether the oviposition deterrent activity of these compounds is to be attributed to the active ingredient (such as the co-formulates) or, as already highlighted for copper, to the antibacterial activity which, eliminating the biofilm on the fruit surface, would make the olives less attractive to the fly [32,51].

The antibacterial activity of these compounds should be thoroughly studied, not only with the aim of using them as egg deterrents, but also their possible use in the promising context of Symbiotic Control (SC) [52] and of symbiosis disruption, opening new possibilities for integrated olive fly management programs. Future work will provide indications to maximize the effect of this approach in an open field context, enabling a new and eco-friendly approach for the control of the olive fly to be developed.

## Figures and Tables

**Table 1:** Substances used in the oviposition assay (no-choice) and choice test.

Active Ingredient (Content) and Formulation	Trade Name	Manufacturer	Olive Production Uses *	Application Rate (g or mL hL^−1^)
Copper oxychloride (3.75%), WG	Neoram^®^	Isagro S.P.A.	fungicide	300
Dodine (52.9%), SC	Syllit^®^ 544 SC	ARYSTA LifeScience Italia S.r.l.	fungicide	165
Mancozeb (75%), WDG	ASPOR WDG	SUMITOMO CHEMICAL S.r.l.	fungicide	320
Pyraclostrobin (20%), WG	Cabrio^®^ WG	BASF Agricultural Solution Italia	fungicide	50
Difeconazole (23.6%), EC	Score^®^ 25 EC	Syngenta Italia S.p.A.	fungicide	50
Tannins (0.13%), SL	Distillato di legno	BioDea	plant biostimulant	200
Clay; clinoptilolite-heulandite (67.5%) + mordenitis (32.5%), WP	Zeolite CUBANA Bio^®^	BioAgrotech S.r.l.	plant biostimulant	400
Flavonoids (2.00%), SL	Propolis serbios	Serbios S.r.l.	plant biostimulant	300
Cu (2%) + Zn (4%) + citric acid (23.8%), SL	Dentamet^®^	DIACHEM S.p.A.	plant biostimulant	547
*Beauveria bassiana* ATCC 74040 (7.16%), OD	Naturalis^®^	BIOGARD^®^	entomopathogenic fungus	200

* used in accordance with Annex I of the new EU Fertilising Products Regulation (EU) 2019/1009 which shall apply from 16 July 2022 and with Annex III to the Regulation (EC) No 1185/2009 concerning statistics on pesticides. SL: soluble concentrate; WP: wettable powder; OD: dispersible oil; WG: water dispersible granule; EC: emulsifiable concentrate; SC: suspension concentrate.

## Data Availability

The data presented in this study are available in article.
